# Author Correction: Structure and topography of the synaptic V-ATPase–synaptophysin complex

**DOI:** 10.1038/s41586-025-09149-x

**Published:** 2025-05-22

**Authors:** Chuchu Wang, Wenhong Jiang, Jeremy Leitz, Kailu Yang, Luis Esquivies, Xing Wang, Xiaotao Shen, Richard G. Held, Daniel J. Adams, Tamara Basta, Lucas Hampton, Ruiqi Jian, Lihua Jiang, Michael H. B. Stowell, Wolfgang Baumeister, Qiang Guo, Axel T. Brunger

**Affiliations:** 1 Department of Molecular and Cellular Physiology, Stanford University, Stanford, CA USA; 2 Department of Neurology and Neurological Sciences, Stanford University, Stanford, CA USA; 3 Department of Structural Biology, Stanford University, Stanford, CA USA; 4 Department of Photon Science, Stanford University, Stanford, CA USA; 5 Howard Hughes Medical Institute, Stanford University, Stanford, CA USA; 6 State Key Laboratory of Protein and Plant Gene Research, School of Life Sciences and Peking-Tsinghua Center for Life Sciences, Peking University, Beijing, China; 7 Department of Genetics, Stanford University, Stanford, CA USA; 8 Stanford Center for Genomics and Personalized Medicine, Stanford University, Stanford, CA USA; 9 Lee Kong Chian School of Medicine, Nanyang Technological University, Singapore, Singapore; 10 Department of Molecular, Cellular, and Developmental Biology, University of Colorado, Boulder, CO USA; 11 Department of Structural Biology, Max Planck Institute of Biochemistry, Martinsried, Germany

**Keywords:** Synaptic vesicle exocytosis, Cryoelectron tomography

Correction to: *Nature* 10.1038/s41586-024-07610-x Published online 5 June 2024

In the version of the article initially published, in the second paragraph of the “Isolation of glutamatergic synaptic vesicles” section of the Methods, the catalogue number of the VGLUT1 monoclonal antibody was listed as “(135303, SySy)” and has now been corrected to “(135311, SySy)”. In the second paragraph of the “SDS–PAGE and western blotting” section of the Methods, the antibody name “ATP6V1A1” has been corrected to “ATP6V0A1”. These corrections have been made to the HTML and PDF versions of the article.

