# Supplementary material for: Structure and topography of the synaptic V-ATPase–synaptophysin complex
Source: Nature. 2024 Jun 5;631(8022):899–904. doi: 10.1038/s41586-024-07610-x (PMC11269182; doi:10.1038/s41586-024-07610-x)
Supplement: Supplementary file 1 — This file contains Supplementary Tables 2–5 [file 41586_2024_7610_MOESM1_ESM.pdf]

nature portfolio

https://doi.org/10.1038/s41586-024-07610-x

# Supplementary information

# Structure and topography of the synaptic V-ATPase–synaptophysin complex

In the format provided by the authors and unedited

**Supplementary Information**

**Structure and Topography of the Synaptic V-ATPase–Synaptophysin Complex**

Chuchu Wang[1-5†], Wenhong Jiang[6†], Jeremy Leitz[1-5†], Kailu Yang[1-5], Luis Esquivies[1-5], Xing Wang[6], Xiaotao Shen[7,8], Richard Held[1-5], Daniel J. Adams[9], Tamara Basta[9], Lucas Hampton[9], Ruiqi Jian[7,8], Lihua Jiang[7,8], Michael H.B. Stowell[9], Wolfgang Baumeister[10], Qiang Guo[6$], Axel T. Brunger[1-5$]

[1]Department of Molecular and Cellular Physiology, Stanford University, Stanford, United States

[2]Department of Neurology and Neurological Sciences, Stanford University, Stanford, United States

[3]Department of Structural Biology, Stanford University, Stanford, United States

[4]Department of Photon Science, Stanford University, Stanford, United States

[5]Howard Hughes Medical Institute, Stanford University, Stanford, United States

[6]State Key Laboratory of Protein and Plant Gene Research, School of Life Sciences and Peking-Tsinghua Center for Life Sciences, Peking University, Beijing 100871, China

[7]Department of Genetics, Stanford University, Stanford, United States.

[8]Stanford Center for Genomics and Personalized Medicine, Stanford University, Stanford, United States.

[9]Department of Molecular, Cellular, and Developmental Biology, University of Colorado, Boulder, CO 80309

[10]Department of Structural Biology, Max Planck Institute of Biochemistry, 82152

[†]These authors contributed equally

[$]Correspondence: brunger@stanford.edu, guo.qiang@pku.edu.cn

**Table of contents**

**Page**

**Supplementary Table 1 –** List of wild-type ISV proteins identified by high-resolution mass-spectrometry with map cross-correlation scores. Separate Excel file (SupplementaryTable1.xlsx)

**Supplementary Table 2 –** Identification of unique peptides of the V-ATPase subunit e2 from wild-type ISV samples. 3

**Supplementary Table 3 –** Root-mean square differences between V-ATPase structures. 3

**Supplementary Table 4 –** Copy numbers of intact V-ATPase assemblies per ISV. 3

**Supplementary Table 5 –** Copy numbers of V0-only V-ATPase assemblies per ISV. 4

**Supplementary Table 2.** Identification of unique peptides of the V-ATPase subunit e2 from wild-type ISV samples

| Identified protein | Biological replicate | Score | Unique peptide[1] |
|---|---|---|---|
| | ISV-1 | 307.7 | Y.VRFLWE. |
| | ISV-2 | 308.8 | F.TTF.I |
| ATP6V0E2 | ISV-3 | 303.7 | F.TTF.W |

[1]The spectrum score uses Byonic's scoring algorithm.

**Supplementary Table 3.** Root-mean-square differences between V-ATPase structures

| RMSD (Å) | V-ATPase | In wild-type ISVs | | | |
|---|---|---|---|---|---|
| | | State 1 | State 2 | State 3 | V0-only |
| In $Syp^{-/-}$ ISVs | State 1 | 2.3 | | | |
| | State 2 | | 2.1 | | |
| | State 3 | | | 1 | |
| | V0-only | | | | 1.7 |
| PDB IDs of other State 2 | 6WM3 | | 3.3 | | |
| | 7U4T | | 3.8 | | |
| | 6VQG | | 4 | | |
| | 7UNF | | 4.1 | | |

Structures were globally aligned by superimposing Cα atoms and root-mean-square differences (RMSDs) calculated for Cα atoms. State 2 was used for comparisons of wild-type intact V-ATPase with the other mammalian structures (PDB IDs: 6WM3, 7U4T, 6VQG, 7UNF) since the 7UNF deposition only includes State 2.

**Supplementary Table 4.** Copy numbers of intact V-ATPase assemblies

| | Number of ISVs | | Percentage[1] | |
|---|---|---|---|---|
| **Intact V-ATPase copy number** | **wild-type** | **$Syp^{-/-}$** | **wild-type** | **$Syp^{-/-}$** |
| 0 | 2228 | 927 | | |
| 1 | 945 | 688 | **71.3** | **47.4** |
| 2 | 290 | 419 | **21.9** | **28.8** |
| 3 | 72 | 218 | **5.4** | **15.0** |
| 4 | 15 | 79 | **1.1** | **5.4** |
| 5 | 4 | 28 | **0.3** | **1.9** |
| 6 | 0 | 11 | **0.0** | **0.8** |
| 7 | 0 | 6 | **0.0** | **0.4** |
| 8 | 0 | 4 | **0.0** | **0.3** |
| **Total examined number of ISVs** | **3554** | **2380** | | |
| **Number of ISVs with at least one intact V-ATPase** | **1326** | **1453** | | |

[1]Normalized to the number ISVs with at least one intact V-ATPase

**Supplementary Table 5.** Copy numbers of V0-only V-ATPase assemblies

| | Number of ISVs | | Percentage[1] | |
|---|---|---|---|---|
| **V0-only V-ATPase copy number** | **wildtype** | **$Syp^{-/-}$** | **wild-type** | **$Syp^{-/-}$** |
| 0 | 197 | 233 | | |
| 1 | 86 | 130 | **81.1** | **69.1** |
| 2 | 18 | 46 | **17.0** | **24.5** |
| 3 | 2 | 11 | **1.9** | **5.9** |
| 4 | 0 | 1 | **0.0** | **0.5** |
| **Total examined number of ISVs** | **303** | **421** | | |
| **Number of ISVs with at least one V0-only V-ATPase** | **106** | **188** | | |

[1]Normalized to the number ISVs with at least one V0-only V-ATPase.
