# Supplementary material for: Structure and topography of the synaptic V-ATPase–synaptophysin complex
Source: Nature. 2024 Jun 5;631(8022):899–904. doi: 10.1038/s41586-024-07610-x (PMC11269182; doi:10.1038/s41586-024-07610-x)
Supplement: Supplementary file 2 — Reporting Summary [file 41586_2024_7610_MOESM2_ESM.pdf]

# nature portfolio

Corresponding author(s): Axel T. Brunger

Last updated by author(s): May 13, 2024

# Reporting Summary

Nature Portfolio wishes to improve the reproducibility of the work that we publish. This form provides structure for consistency and transparency in reporting. For further information on Nature Portfolio policies, see our Editorial Policies and the Editorial Policy Checklist.

## Statistics

For all statistical analyses, confirm that the following items are present in the figure legend, table legend, main text, or Methods section.

| n/a | Confirmed | |
|---|---|---|
| ☐ | ☒ | The exact sample size (*n*) for each experimental group/condition, given as a discrete number and unit of measurement |
| ☐ | ☒ | A statement on whether measurements were taken from distinct samples or whether the same sample was measured repeatedly |
| ☐ | ☒ | The statistical test(s) used AND whether they are one- or two-sided<br>*Only common tests should be described solely by name; describe more complex techniques in the Methods section.* |
| ☒ | ☐ | A description of all covariates tested |
| ☐ | ☒ | A description of any assumptions or corrections, such as tests of normality and adjustment for multiple comparisons |
| ☐ | ☒ | A full description of the statistical parameters including central tendency (e.g. means) or other basic estimates (e.g. regression coefficient) AND variation (e.g. standard deviation) or associated estimates of uncertainty (e.g. confidence intervals) |
| ☐ | ☒ | For null hypothesis testing, the test statistic (e.g. *F*, *t*, *r*) with confidence intervals, effect sizes, degrees of freedom and *P* value noted<br>*Give P values as exact values whenever suitable.* |
| ☒ | ☐ | For Bayesian analysis, information on the choice of priors and Markov chain Monte Carlo settings |
| ☒ | ☐ | For hierarchical and complex designs, identification of the appropriate level for tests and full reporting of outcomes |
| ☒ | ☐ | Estimates of effect sizes (e.g. Cohen's *d*, Pearson's *r*), indicating how they were calculated |

*Our web collection on statistics for biologists contains articles on many of the points above.*

## Software and code

Policy information about availability of computer code

| | |
|---|---|
| Data collection | iBright1500 imaging system, SerialEM v4.0&v4.1, Thermo Scientific Xcalibur v4.1 |
| Data analysis | AlphaFold2, Amira v2020.2, AreTomo v1.3.4, Byonic by ProteinMetrics v5.1.1, Chimera v1.16 & ChimeraX v1.3&1.7, cryoSPARC v4.4, CTFFIND4, iBright1500 Analysis Software, IMOD v 4.11.3, ISOLDE v1.3, Motioncor2, Phenix v1.21, EMRinger v1.0.0, Prism v10.2.2 (GraphPad Software), Proteome Discoverer v2.1 (Thermo Fisher Scientific), PyCrESTA (https://github.com/brungerlab/pycresta), PyMol v2.3.2 (Schrödinger, LLC), Python v3.8, PySeg v1.0.0, PyTom v0.981a&1.1, R v3.4.2, RELION v3.1&4.0, Rosetta 2, Scientific Xcalibur (v4.1, Thermo Fisher Scientific), SerialEM v4.0&4.1, Situs v3.2, Topaz v0.2.5, Warp v1.0.9. Several script files are available at https://github.com/brungerlab/ISV_scripts. |

For manuscripts utilizing custom algorithms or software that are central to the research but not yet described in published literature, software must be made available to editors and reviewers. We strongly encourage code deposition in a community repository (e.g. GitHub). See the Nature Portfolio guidelines for submitting code & software for further information.

## Data

Policy information about availability of data

All manuscripts must include a data availability statement. This statement should provide the following information, where applicable:

- Accession codes, unique identifiers, or web links for publicly available datasets
- A description of any restrictions on data availability
- For clinical datasets or third party data, please ensure that the statement adheres to our policy

The subtomogram averaging maps (wild-type V0-only: 44858, wild-type State 1: 44855, wild-type State 2: 44856, wild-type State 3: 44857), the SPA maps (wild-type V0-only: 44846, wild-type State 1: 44843, wild-type State 2: 44839, wild-type State 3: 44840, Syp-/- V0-only: 44845, Syp-/- State 1: 44844, Syp-/- State 2: 44842, Syp-/- State 3: 44841) and representative binned tomograms (Syp-/- ISV:44847, wild-type ISV:44848) have been deposited in the EMDB, and the atomic coordinates have been deposited in the PDB (wild-type V0-only: 9BRZ, wild-type State 1: 9BRT, wild-type State 2: 9BRA, wild-type State 3: 9BRQ, Syp-/- V0-only: 9BRY, Syp-/- State 1: 9BRU, Syp-/- State 2: 9BRS, Syp-/- State 3: 9BRR).

## Research involving human participants, their data, or biological material

Policy information about studies with human participants or human data. See also policy information about sex, gender (identity/presentation), and sexual orientation and race, ethnicity and racism.

| | |
|---|---|
| Reporting on sex and gender | N/A |
| Reporting on race, ethnicity, or other socially relevant groupings | N/A |
| Population characteristics | N/A |
| Recruitment | N/A |
| Ethics oversight | N/A |

Note that full information on the approval of the study protocol must also be provided in the manuscript.

# Field-specific reporting

Please select the one below that is the best fit for your research. If you are not sure, read the appropriate sections before making your selection.

☒ Life sciences ☐ Behavioural & social sciences ☐ Ecological, evolutionary & environmental sciences

For a reference copy of the document with all sections, see nature.com/documents/nr-reporting-summary-flat.pdf

# Life sciences study design

All studies must disclose on these points even when the disclosure is negative.

| | |
|---|---|
| Sample size | Experiments described in this study were performed with at least 3-9 samples for each group, which is consistent with sample sizes commonly used in similar studies in the field. And the sample sizes provided adequate power to detect significant effects. |
| Data exclusions | Since it is difficult to determine the true number of ISVs without any V-ATPase assembly due to the missing wedge effect of tomographic reconstructions, we fitted the Poisson distributions to copy numbers ≥ 1. |
| Replication | All successful data were generated over multiple attempts and in at least three replicates (replicates number are specified in the manuscript) |
| Randomization | Experiments randomization was not necessary, but in all experiments, control and experimental samples were analyzed in parallel. |
| Blinding | Two individuals independently examined two halves of the tomograms and counted the copy numbers of V-ATPase per ISV. The two individuals knew the samples of the dataset, but didn't know each other's counting results. The mice observational behavior was scored blind by two individuals. |

# Reporting for specific materials, systems and methods

We require information from authors about some types of materials, experimental systems and methods used in many studies. Here, indicate whether each material, system or method listed is relevant to your study. If you are not sure if a list item applies to your research, read the appropriate section before selecting a response.

## Materials & experimental systems

| n/a | Involved in the study |
|---|---|
| ☐ | ☒ Antibodies |
| ☒ | ☐ Eukaryotic cell lines |
| ☒ | ☐ Palaeontology and archaeology |
| ☐ | ☒ Animals and other organisms |
| ☒ | ☐ Clinical data |
| ☒ | ☐ Dual use research of concern |
| ☒ | ☐ Plants |

## Methods

| n/a | Involved in the study |
|---|---|
| ☒ | ☐ ChIP-seq |
| ☒ | ☐ Flow cytometry |
| ☒ | ☐ MRI-based neuroimaging |

# Antibodies

Antibodies used

mouse-VGLUT1(1:200, SySy, Cat.# 135303); rabbit-synaptophysin-1 (1:1,000; SySy, Cat.# 101008); rabbit-IRDye800CW (1:3,000; LI-COR, Cat.# 926-32211); mouse-synaptobrevin-2 (1:1,000; SySy, Cat.# 104211); mouse-HRP (1:10,000; Abcam, Cat.# ab6789); mouse-synaptotagmin-1 (1:1,000; SySy, Cat.# 105011); mouse-IRDye800CW (L1:3,000; I-COR, Cat.# 926-32210); rabbit-synaptoporin-1 (1:500; SySy, Cat.# 102002); rabbit-ATP6V1A1 (1:1,000; NovusBio, Cat.# NBP1-89342); rabbit-synaptogyrin-1 (1:500; SySy, Cat.# 103002); rabbit-VGLUT1 (1:1,000; SySy, Cat.# 135303);

Validation

mouse-VGLUT1 validated PubMed: 15103023; rabbit-synaptophysin-1 is validated by the company; mouse-synaptobrevin-2 validated PubMed: 26663078; mouse-synaptotagmin-1 validated PubMed: 29274147; rabbit-synaptoporin-1 validated PubMed: 31090538; rabbit-ATP6V1A1 validated of the orthogonal strategies by the company ; rabbit-synaptogyrin-1 validated PubMed: 31090538; rabbit-VGLUT1 validated PubMed: 15103023.

# Animals and other research organisms

Policy information about studies involving animals; ARRIVE guidelines recommended for reporting animal research, and Sex and Gender in Research

Laboratory animals

Male wild-type CD1 mice (23-26 days old) and C57BL6 mice (23-26 days old) were used for synaptic vesicle preparations. Male Syp -/- mice (23-26 days old) were used for synaptic vesicle preparations. Both sexes of 4-6 months-old wild-type Black 6 (B6NTac) and Syp-/- mice were used in animal behavior experiments.

Wild animals

N/A

Reporting on sex

Sexes were reported above

Field-collected samples

N/A

Ethics oversight

All animal procedures were performed in accordance with the National Institutes of Health Guide for the Care and Use of Laboratory Animals and approved by the Stanford Administrative Panel on Laboratory Animal Care (APLAC) institutional guidelines (protocol #29981) and by the University of Colorado Boulder Institutional Animal Care and Use Committee (IACUC) (protocol #1106.02). No field collected samples were used in the study.

Note that full information on the approval of the study protocol must also be provided in the manuscript.

# Plants

Seed stocks

N/A

Novel plant genotypes

N/A

Authentication

N/A
